# Cross-sectional associations between weight-related health behaviors and weight misperception among U.S. adolescents with overweight/obesity

**DOI:** 10.1186/s12889-018-5394-9

**Published:** 2018-04-18

**Authors:** Samantha L. Hahn, Kelley A. Borton, Kendrin R. Sonneville

**Affiliations:** Department of Nutritional Sciences, University of Michigan School of Public Health, 1415 Washington Heights, Ann Arbor, MI 48109 USA

**Keywords:** Weight misperception, Adolescent, Overweight, Obesity, Physical activity, Nutrition

## Abstract

**Background:**

Weight misperception occurs when there is a discrepancy between one’s actual and perceived weight status. Among adolescents with overweight/obesity, many believe that correcting weight misperception is imperative to inspire weight-related behavior change. However, past research has shown that adolescents with overweight/obesity who misperceive their weight status gain less weight over time compared to accurate perceivers. Therefore, our objective was to examine possible mechanisms underlying this relationship. Specifically, we examined the association between weight misperception and engagement in weight-related health behaviors among adolescents with overweight/obesity.

**Methods:**

Self-reported data from the 2015 National Youth Risk Behavior Survey was used in analyses restricted to participants with overweight/obesity (*n* = 4383). Using multivariate logistic models correcting for sex, race/ethnicity, and grade in school, we examined the cross-sectional associations between weight misperception and engagement in weight-related health behaviors, specifically related to dietary intake, physical activity, and sleep.

**Results:**

Adolescents with overweight/obesity who misperceived their weight status were more likely to drink 100% fruit juice two or more times per day (OR = 1.53, 95% CI: 1.20, 1.94), eat vegetables two or more times per day (OR = 1.29, 95% CI: 1.07, 1.57), be physically active for 1 hour or more per day for at least 5 days in the week prior (OR = 1.40, 95% CI: 1.15, 1.72), be on a sports team in the last year (OR = 1.55, 95% CI: 1.21, 1.97), sleep an average of at least 8 hours per school night (OR = 1.40, 95% CI: 1.15, 1.72), and less likely to be trying to lose weight (OR = 0.17, 95% CI: 0.15, 0.20). Misperceivers were more likely to consume breakfast every morning in the week prior and to drink a sports drink at least once per day, though these results were not statistically significant. We observed no difference in fruit intake, soda intake, or TV viewing between weight misperceivers and accurate perceivers.

**Conclusions:**

Overall, weight misperception among adolescents with overweight/obesity was associated with a number of beneficial weight-related health behaviors. Engagement in these healthy weight-related behaviors may explain some of the protective effect of weight misperception on weight gain over time.

**Trial registration:**

Not applicable.

## Background

The prevalence of overweight and obesity among adolescents is at an all-time high in the United States [1]. Adolescents with obesity are at increased risk for a number of adverse health conditions, both physical and mental, including elevated blood pressure and cholesterol, stroke, heart attack, depression, body dissatisfaction, binge eating, and engagement in disordered weight control behaviors [2–6]. Because of the numerous physical and mental health consequences associated with obesity, identifying strategies to prevent and treat excess weight gain has become a top priority in public health.

One area of particular interest in the prevention and treatment of obesity is the correction of weight misperception among individuals with overweight/obesity. Weight misperception, the phenomenon that occurs when there is a discrepancy between one’s perceived weight status and their actual weight status, is particularly common among adolescents with overweight/obesity. Among children and adolescents in the United States, 77% of those with overweight, and 43% of those with obesity, misperceive their weight to be within the healthy range [7]. Weight misperception among youth with overweight/obesity is more common among boys, those from low-income families, and racial/ethnic minorities [7–9]. Despite the increase in prevalence of overweight/obesity among adolescents, the proportion of those adolescents who misperceive their weight status has remained stable [10, 11].

Some have argued that it in order to inspire weight-related behavior change among adolescents with overweight/obesity, it is necessary to correct weight misperception [7, 12, 13]. However, the evidence to support this belief is conflicting. Some studies have found that individuals that accurately perceive their weight status are more likely to report engaging in healthy behaviors [13–15]. Conversely, compared to weight misperceivers, adolescents and young adults that accurately perceived their weight status are at increased odds of engaging in disordered weight control behaviors and binge eating [16–19], which are independently associated with future weight gain [20–22]. Individuals with overweight/obesity who accurately perceive their weight status are also less likely to meet recommendations for fruit and vegetable consumption, and physical activity [14]. Additionally, longitudinal data show that among adolescents with overweight/obesity, those who misperceive themselves to be of a normal weight gain less weight over time compared to accurate perceivers, though the mechanism is unclear [23, 24]. Understanding how weight-related behaviors differ between adolescents with and without weight misperception is an important first step in elucidating these mechanisms and the evidence thus far has been conflicting. Therefore, in an effort to better understand why adolescents with overweight/obesity who misperceive their weight status gain less weight over time compared to accurate perceivers, we aim to examine the associations between weight misperception and weight-related health behaviors including dietary intake, physical activity, and sleep, among a nationally representative sample of high school adolescents with overweight/obesity.

## Methods

### Study design and population

Data for this study came from the 2015 National Youth Risk Behavior Survey (YRBS). The YRBS is a cross-sectional school-based survey conducted by the Center for Disease Control and Prevention (CDC) in order to monitor priority health behaviors among adolescents nationwide [25]. The survey monitors behaviors that are associated with the leading causes of death and disability among youth, including both healthy and unhealthy diet-related behaviors and physical activity. The YRBS employs a three-stage cluster sample design and is administered to a sample of adolescents enrolled in grades 9 through 12 in both public and private schools, making the data nationally representative of high school students. Participation is the YRBS is voluntary. Parental consent is obtained according to local procedure prior to students completing the anonymous self-administered surveys. Further information on the specifics of the sampling methods and survey development can be found elsewhere [25].

For the purpose of this study, analyses were restricted to respondents with overweight/obesity based on self-reported height and weight. Participants were classified as having overweight/obesity if their BMI was at or above the 85th percentile for age- and sex-specific BMI growth charts. Individuals with overweight/obesity were combined into a single category to remain consistent with prior research examining weight misperception among individuals with overweight/obesity [8–12, 14, 16, 19, 23, 26–29]. Of the original 14,358 adolescents surveyed during the 2015 YRBS who completed height and weight questions, 31.6% (*n* = 4536) were categorized as having overweight/obesity based on their self-reported height and weight. After excluding participants who were not enrolled in grades 9 through 12, had missing data for sex, race/ethnicity, or weight perception, our final study population consisted of 4383 adolescents with overweight/obesity.

### Study variables

#### Weight misperception

Weight misperception among participants with overweight/obesity was determined using respondents’ answer to the following question: “How do you describe your weight?” Possible responses were: very underweight, slightly underweight, about the right weight, slightly overweight, and very overweight. Participants who answered ‘very underweight’, ‘slightly underweight’, or ‘about the right weight’ were considered weight misperceivers. Respondents who answered ‘slightly overweight’ or ‘very overweight’ were categorized as accurate weight perceivers. Individuals with obesity who answered ‘slightly overweight’ were considered accurate perceivers despite some level of misperception because they identified themselves to be of the overweight category. It has been theorized that identifying as part of this highly stigmatized group may be what determines psychological well-being and engagement in behaviors that ultimately affect weight [30].

#### Weight-related health behaviors

The outcome of interest for this study was engagement in various weight-related health behaviors, specifically related to dietary intake, physical activity, and sleep. We examined fruit and fruit juice consumption, vegetable consumption, regularly eating breakfast, drinking soda, drinking sports drinks, being physically active, participating on a sports team, watching TV, sleep, and trying to lose weight. Survey questions pertaining to nutritional behaviors included, “During the past 7 days, how many times did you drink 100% fruit juices such as orange juice, apple juice, or grape juice?”, “During the past 7 days, how many times did you eat fruit?”, “During the past 7 days, how many times did you drink a can, bottle, or glass of soda or pop, such as Coke, Pepsi, or Sprite? (Do not count diet soda or diet pop).” For these questions, possible responses included: I did not, 1 to 3 times during the past 7 days, 4 to 6 times during the past 7 days, 1 time per day, 2 times per day, 3 times per day, and 4 or more times per day. Additionally, we assessed breakfast consumption using the survey question, “During the past 7 days, on how many days did you eat breakfast?” Participants had the option of selecting an integer from 0 to 7. Vegetable consumption was ascertained using four separate questions asking about green salad, potatoes, carrots, and other vegetables. One example of these questions was, “During the past 7 days, how many times did you eat carrots?” With answer options of: I did not eat carrots during the past 7 days, 1 to 3 times during the past 7 days, 4 to 6 times during the past 7 days, 1 time per day, 2 times per day, 3 times per day, and 4 or more times per day. The four questions pertaining to vegetable consumption were ultimately combined to a single variable based on total number of times eating vegetables per day.

In order to assess physical activity we used the survey question, “During the past 7 days, on how many days were you physically active for a total of at least 60 minutes per day?” Participants again had the option of selecting an integer from 0 to 7. In addition, we also examined physical activity using the question, “During the past 12 months, on how many sports teams did you play? (Count any teams run by your school or community groups)”. Potential answers were: 0 teams, 1 team, 2 teams, or 3 or more teams. To assess television watching, a marker of physical inactivity [31], we used the following question, “On an average school day, how many hours do you watch TV?” Possible answers for this question included I do not, less than 1 h per day, 1 h per day, 2 h per day, 3 h per day, 4 h per day, or 5 or more hours per day.

In addition to these nutrition and physical activity behaviors, we also asked about sleep and weight loss intention. Sleep was assessed using the question, “On an average school night, how many hours of sleep do you get?” with possible answer options of: 4 or less hours, 5 h, 6 h, 7 h, 8 h, 9 h, or 10 or more hours. Weight loss intention was assessed using the question, “Which of the following are you trying to do about your weight”, with response options of: lose weight, gain weight, stay the same weight, or I am not trying to do anything about my weight.

Variables were dichotomized based on cut-offs informed by prior research and expert recommendations. Fruit, fruit juice, and vegetable consumption were each made into dichotomous variables indicating consumption two or more times per day versus less than two times per day. This cutoff was chosen based on the general recommendation of five servings of fruits and vegetables per day and the most recent set of Dietary Guidelines for Americans which recommends consuming at least two cup equivalents of fruit and two and a half cup equivalents of vegetables [32]. Breakfast was categorized as breakfast all seven days in the week prior to being surveyed versus less than every day based on expert recommendation, and the evidence suggesting that skipping breakfast was associated with increased risk of overweight/obesity and BMI gain among adolescents [33–35]. Soda and sports drink consumption were each dichotomized into less than once per day compared to once or more per day in order to compare regular consumption versus less regular consumption, as well as to allow our results to be compared to existing literature examining sugar sweetened beverage consumption [36, 37]. Physical activity was dichotomized to at least five days in the week prior to survey compared to less than five days of sixty or more minutes of physical activity. Although the recommendation is for adolescents to get sixty minutes of physical activity every day [32], in order to be able to compare our results with others using YRBS data and to examine those who are regularly engaging in physical activity, we elected to dichotomize at five or more days in the past week [26, 38]. Playing on a sports team was made into a binary variable representing any or none based on prior research indicating that engagement in sports teams is associated with decreased risk for overweight/obesity among adolescents, as well as associated with a number of beneficial psychological and social outcomes [39, 40]. A dichotomous variable for TV viewing was created using a cut-off of three or more hours versus two or less hours based on recommendations for adolescents to have two or fewer hours of screen time per day [35]. Sleep was assessed using an average of eight or more hours of sleep per school night compared to less than eight hours of sleep per night based on recommendations for adolescents to get at least eight hours of sleep per night [41]. Participants were categorized as trying to lose weight or not trying to lose weight. When asked what they were trying to do about their weight, those that answered that they were trying to gain weight, stay the same weight, or were not trying to do anything about their weight were combined into one group who were ‘not trying to lose weight’ compared to those that answered that they were trying to lose weight.

### Demographics

Sex, race/ethnicity, and grade in school were all included as covariates in the statistical models and were based on the self-reported responses. Race/ethnicity classifications were based on answers to the following questions: “What is your race? (Select one or more responses)” and, “Are you Hispanic or Latino?” For analyses, respondents were categorized as non-Hispanic white, non-Hispanic black/African American, Hispanic/Latino (including individuals who identified as multiple race/ethnicities but also identified as Hispanic/Latino), or all other races/ethnicities (including American Indian/Alaskan Native, Asian, Native Hawaiian/Other Pacific Islander, or non-Hispanic multiple race/ethnicities).

### Statistical analysis

All analyses were conducted using SAS 9.4 and took into account the complex sampling design employed in the YRBS data collection process. Individual adjusted logistic regression models were conducted for each of the outcomes of interest with weight misperception as the primary exposure of interest and sex, race/ethnicity, and grade in school as covariates. Odds ratios (OR) were calculated and reported with the 95% confidence interval (CI). Results were considered significant for *p*-values ≤0.05.

## Results

### Demographics

Demographic characteristics of our study sample can be found in Table 1, as can prevalence estimates of weight misperception by demographic characteristics. Overall, 31.2% of respondents with overweight/obesity misperceived their weight status. Prevalence estimates of weight misperception in this sample differed by sex, with lower prevalence of weight misperception among females compared to males (*p* < .01). Prevalence estimates of weight misperception also differed by race/ethnicity (*p* < .01). Weight misperception was most prevalent among non-Hispanic black or African American adolescents with overweight/obesity, with 41.0% of these students misperceiving their weight status compared to 37.2% of Native Hawaiian/other Pacific Islander, 31.0% of non-Hispanic white students, 30.7% of American Indian/Alaskan native students, 28.3% of Hispanic/Latino students, 23.3% of students identifying as multiple race/ethnicities and 20.8% of Asian students with overweight/obesity. The prevalence of weight misperception also differed by grade in school with marginal significance (*p* = .08), with prevalence decreasing as grade increased. Among 9th graders surveyed, 35.6% of students misperceiving their weight status, whereas only 27.7% of students in 12th grade misperceived their weight status.

**Table 1:** Prevalence of weight misperception by demographic characteristics among U.S. high school students with overweight/obesity

Demographic characteristics	Sampled frequency, *n* (%)	Weight misperception frequency, *n* (%)
Sex^a^		
Female	2003 (44.5)	426 (21.4)
Male	2380 (55.5)	900 (39.1)
Race/ethnicity^b^		
American Indian/Alaskan Native	50 (0.6)	17 (30.7)
Asian	111 (2.2)	33 (20.8)
Hispanic/Latino	1672 (25.3)	457 (28.3)
Multiple Race/Ethnicities Non-Hispanic	223 (5.1)	67 (23.3)
Native Hawaiian/other Pacific Islander	29 (0.6)	8 (37.2)
Non-Hispanic black or African American	479 (14.5)	202 (41.0)
Non-Hispanic white	1819 (51.8)	542 (31.0)
Grade in school^c^		
9th	1120 (26.4)	376 (35.6)
10th	1129 (26.6)	357 (30.7)
11th	1152 (24.3)	330 (30.2)
12th	982 (22.7)	263 (27.7)

All Percentages reported are weighted to account for sampling design

^a^Prevalence estimates of weight misperception and all disordered weight control behaviors differ by sex (*p* < .01)

^b^Prevalence estimates of weight misperception differ by race/ethnicity (*p* < .01)

^c^Prevalence estimates of weight misperception marginally different by grade in school (*p* = .08)

### Associations between weight-related health behaviors and weight misperception

**Fig. 1:**
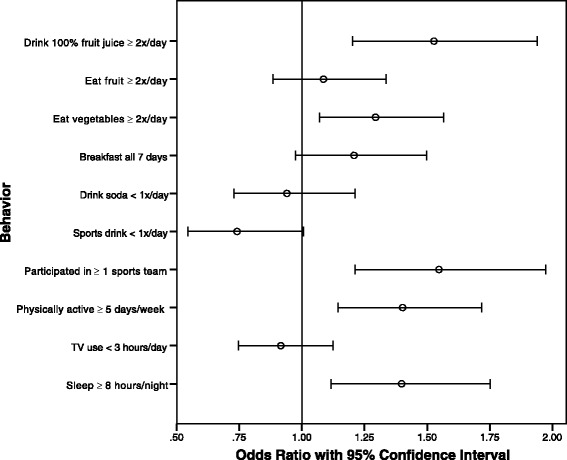
Odds of reporting engagement in weight-related health behaviors among weight misperceivers compared to accurate perceivers

In logistic regression models adjusting for sex, race/ethnicity, and grade in school, weight misperception was associated with a number of the weight-related health behaviors that we examined (Fig. 1). Specifically, weight misperception was associated with increased odds of drinking 100% fruit juice two or more times per day (OR = 1.53, 95% CI: 1.20, 1.94), eating vegetables two or more times per day (OR = 1.29, 95% CI: 1.07, 1.57), being physically active for at least sixty minutes five or more days in the week prior to being surveyed (OR = 1.40, 95% CI: 1.15, 1.72), participating in at least one sports team in the last year (OR = 1.55, 95% CI: 1.21, 1.97), and sleeping an average of at least eight hours on school nights (OR = 1.40, 95% CI: 1.15, 1.72). Those that misperceived their weight status were also more likely to eat breakfast all seven days in the week prior to being surveyed (OR = 1.21, 95% CI: 0.97, 1.50) though this association was not statistically significant. Adolescents who misperceived their weight status were also less likely to drink a sports drink less than once per day (OR = 0.74, 95% CI: 0.55, 1.01), meaning that weight misperceivers were more likely to drink one or more sports drinks per day, although these results were also not significant. There was no apparent association between weight misperception and eating fruit two or more times per day (OR = 1.09, 95% CI: 0.88, 1.34), watching less than three hours of TV per day (OR = 0.92, 95% CI: 0.75, 1.12), or drinking soda less than one time per day (OR = 0.94, 95% CI: 0.73, 1.21). In addition, those who misperceived their weight status were less likely to report trying to lose weight (OR = 0.17, 95% CI: 0.14, 0.21).

## Discussion

In a nationally representative sample of adolescents with overweight/obesity, nearly one-third misperceived their weight status. Weight misperception was more common among males, and among those who identified as non-Hispanic black or African American. Weight misperceivers were more likely to engage in a variety of healthy weight-related health behaviors. Specifically, weight misperceivers were more likely to consume 100% fruit juice and vegetables at least two times per day, to report engaging in physical activity at least 60 minutes five or more days in the week prior to survey, to participate in at least one sports team in the past year, and to achieve an average of eight or more hours of sleep per school night. Additionally, weight misperceivers were less likely to be trying to lose weight. However, those that misperceived their weight status were marginally more likely to report drinking sports beverages one or more times per day and to consume breakfast all 7 days in the week prior to being surveyed. There were no significant associations between weight misperception and eating fruit two or more times per day, watching 3 hours or less of TV per day, or consuming one or more sodas per day.

Findings from the current study are supported by other studies that have shown higher prevalence of weight misperception among males and racial/ethnic minorities [7–9, 15]. While previous studies have found that weight misperceivers have decreased physical activity [12], our findings align with others who have found that weight misperceivers actually have higher levels of certain forms of physical activity compared to accurate perceivers in both adults and adolescents [27, 28]. Other studies among adolescents have examined physical activity for weight loss and have found that weight misperceivers are less likely to report engaging in exercise for weight loss purposes, therefore, it is possible that weight misperceivers exercise more but do not do so with the intention of weight loss [14, 15]. To our knowledge, no other group has examined the relationship between sports drink consumption and weight misperception among adolescents. Our findings that adolescents who misperceive their weight status may be more likely to consume sports drinks one or more times per day, could be due to increased physical activity and sport engagement, which has been shown to be associated with increased sports drink consumption [42]. In agreement with findings from the present study, Hsu, et al. found that Taiwanese students aged 10-18 with overweight/obesity who misperceived their weight status were more likely to engage in physical activity, and sleep more than eight hours per day [29]. Similar to our own findings, Hsu, et al. also found that there was no difference between weight misperceivers and accurate perceivers for daily screen time or soft drink consumption [29]. However, in contrast to our findings among students from the United States, among Taiwanese students, weight misperceivers were less likely to eat breakfast every day and there was no difference in fruit and vegetable consumption between weight misperceivers and accurate perceivers [29]. It is possible that these differences may be cultural. Previous studies using YRBS data have similarly found that male weight misperceivers were more likely to get 60 or more minutes of physical activity at least 5 days in the week prior to survey, and consume at least five servings of fruits and vegetables per day in the week prior to survey [14, 26].

In support of the present findings, a number of studies have also found that among adolescents with overweight/obesity who misperceive their weight status are less likely to report trying to lose weight [14, 15, 26, 28]. Having lower odds of reporting trying to lose weight may be beneficial due to the numerous negative health consequences associated with intentional weight loss or dieting including increased risk for disordered eating, development of an eating disorder and larger increases in BMI [20, 43–45]. Our findings suggest that reporting trying to lose weight may be a poor proxy for measuring whether or not adolescents are engaging in healthy weight-related behaviors. In totality, our findings coincide with past research suggesting that adolescents with overweight/obesity who misperceive their weight status are more likely to report engaging in positive health behaviors associated with weight management, despite being less likely to endorse actively trying to lose weight.

This study had several strengths in its methodology; one key strength being the ability to examine associations in a large nationally representative sample of high school students in the United States. In addition, we analyzed a wide array of weight-related health behaviors that included numerous measures of dietary intake, physical activity, and other behaviors associated with better weight control including adequate sleep and daily breakfast consumption [33–35, 46]. However, the methodology was not without limitations. YRBS data is cross-sectional, and therefore we were not able to demonstrate causality or provide information about the associations between weight misperception and weight-related health behaviors over time. Single item measures were also used to measure weight-related health behaviors, which provide a narrow picture of these behaviors, as does the dichotomization of these variables. Moreover, the only aspect of sleep examined in the current study was sleep duration, as YRBS did not ask questions pertaining to other important aspects of sleep such as sleep quality or sleep related disorders, which may be of importance and warrants further research. Furthermore, we restricted our sample to youth with overweight/obesity based on self-reported height and weight. A CDC study of the validity of self-reported height and weight in the YRBS found that high school students were over-reporting their height and underreporting their weight, which translates to a likely underestimation of overweight/obesity in the YRBS sample [47]. Because self-reported height and weight in adolescents with overweight/obesity is also known to produce a lower estimate of weight misperception compared to measured height and weight measurements, we expect some misclassification of weight misperception in our sample [48].

## Conclusions

In the current study, adolescents with overweight/obesity who misperceived their weight status were more likely than their accurate perceiving peers to engage in a variety of beneficial weight-related health behaviors related to dietary intake, physical activity, and sleep. These associations highlight that despite reporting less intention to lose weight, weight misperceivers were more likely to endorse behaviors that are associated with good health and weight control. Taken with previous research, these results may challenge the standing belief that, among individuals with overweight/obesity, knowledge of weight status is necessary for behavior change, and calls into question the efficacy of strategies that involve informing individuals of their weight status. Longitudinal research is needed to determine if these associations persist over time and if these behaviors are associated with better weight control within weight misperceivers.

## Abbreviations

CDC: Center for Disease Control and Prevention
CI: Confidence Interval
OR: Odds Ratio
YRBS: Youth Risk Behavior Survey
